# Supplementary material for: No mass extinction for land plants at the Permian–Triassic transition
Source: Nat Commun. 2019 Jan 23;10:384. doi: 10.1038/s41467-018-07945-w (PMC6344494; doi:10.1038/s41467-018-07945-w)
Supplement: Supplementary file 1 — Supplementary Information [file 41467_2018_7945_MOESM1_ESM.pdf]

Supplementary Information

No mass extinction for land plants at the Permian–Triassic transition

Nowak et al.

Supplementary Note 1

Literary sources used for diversity analysis pertaining to land plant macrofossils[1-162] and sporomorphs[154,162-249] are listed in the Supplementary References. In addition, unpublished occurrence data from palaeobotanical collections at the institutions listed in Supplementary Table 1 was used for macrofossil diversity analysis.

Supplementary Table 1 – Collections providing unpublished data

collection	city	country
Geological Survey of Austria	Vienna	Austria
Naturhistorisches Museum Wien	Vienna	Austria
Národni Museum - Natural History Museum	Prague	Czech Republic
Museum für Naturkunde - Leibniz-Institut für Evolutions- und Biodiversitätsforschung	Berlin	Germany
Goldfuß-Museum - Steinmann Institut der Universität Bonn	Bonn	Germany
TU Bergakademie Freiberg	Freiberg	Germany
Geowissenschaftliches Museum der Georg-August-Universität Göttingen	Göttingen	Germany
Martin Luther Universität Halle-Wittenberg	Halle	Germany
Bayerische Staatssammlung für Paläontologie und Geologie	Munich	Germany
Bayerisches Landesamt für Umwelt	Munich	Germany
Naturhistorisches Museum Schloss Bertholdsburg	Schleusingen	Germany
Stuttgart State Museum of Natural History	Stuttgart	Germany
Paläontologische Sammlung der Universität Tübingen	Tübingen	Germany
Museo di Paleontologia e Preistoria P. Leonardi	Ferrara	Italy
University of Padova	Padova	Italy
Naturalis Biodiversity Center	Leiden	Netherlands
Laboratory of Palaeobotany and Palynology - Utrecht University	Utrecht	Netherlands
Museo Nacional de Ciencias Naturales	Madrid	Spain
Naturhistoriska riksmuseet	Stockholm	Sweden
Lapworth Museum of Geology	Birmingham	UK
Natural History Museum	London	UK

Supplementary References

1. Abu Hamad, A., Kerp, H., Vörding, B. & Bandel, K. A Late Permian flora with *Dicroidium* from the Dead Sea region, Jordan. *Rev. Palaeobot. Palynol.* **149,** 85–130 (2008).
2. Accordi, B. Resto di conifera nell'Anisico delle Dolomiti. *Riv. Ital. Paleontol. Stratigr.* **58,** (1952).
3. Adendorff, R., Bamford, M. K. & McLoughlin, S. *Liknopetalon*: a review of a rare Gondwanan, Permian pteridophyte. *Rev. Palaeobot. Palynol.* **126,** 83–101 (2003).
4. Alberti, F. von. Beitrag zu einer Monographie des Bunten Sandsteins, Muschelkalks und Keupers und die Verbindung dieser Gebilde zu einer Formation. (Verlag der Gotta'schen Buchhandlung, 1834).
5. Anderson, J. M. & Anderson, H. M. Molteno ferns: Late Triassic biodiversity in southern Africa. *Strelitzia* **21,** (2008).
6. Archangelsky, S. Studies on Triassic fossil plants from Argentins. IV. The leaf genus *Dicroidium* and its possible relation to *Rhexoxylon* stems. *Palaentology* **11,** 500–512 (1968).
7. Archangelsky, S. & Cuneo, R. Ferugliocladaceae, a new conifer family from the Permian of Gondwana. *Rev. Palaeobot. Palynol.* **51,** 3–30 (1987).
8. Arndt, S. Morphologie und Systematik ausgewählter Mesozoischer Koniferen. *Palaeontogr. Abt. B* **262,** 1–23 (2002).
9. Artabe, A. & Brea, M. A new approach to Corystospermales based on Triassic permineralized stems from Argentina. *Alcheringa* **27,** 209–229 (2003).
10. Artabe, A. E., Morel, E. M. & Spalletti, L. A. Paleoecología de las floras triásicas argentinas. in *El Sistema Triásico en la Argentina* (eds. Artabe, A. E., Morel, E. M. & Zamuner, A. B.) 199–225 (Fundación Museo de La Plata 'Francisco Pascasio Moreno', 2001).
11. Artabe, A. E., Morel, E. M., Ganuza, D. G., Zavattieri, A. M. & Spalletti, L. A. The Triassic paleoflora of Potrerillos, Mendoza Province, Argentina. *Ameghiniana* **44,** 279–301 (2007).
12. Artabe, A. E., Brea, M. & Zamuner, A. B. *Rhexoxylon brunoi* Artabe, Brea et Zamuner, sp. nov., a new Triassic corystosperm from the Paramillo de Uspallata, Mendoza, Argentina. *Rev. Palaeobot. Palynol.* **105,** 63–74 (1999).
13. Artabe, A. E. & Stevenson, D. W. Fossil Cycadales of Argentina. *Bot. Rev.* **65,** 219–238 (1999).
14. Asama, K. Permian plants from Maiya, Japan. *Bull. Natl. Sci. Mus. Tokyo Ser. C* **7,** 1–14 (1981).
15. Asama, K. *et al.* Summary of the Carboniferous and Permian Plants from Thailand, Malaysia and Adjacents Areas. in *Geology and Palaeontology of Southeast Asia* (eds. Kobayashi, T. & Toriyama, R.) 77–101 (1975).
16. Asama, K. & Murata, M. Permian plants from Setamai, Japan. *Bull. Natl. Sci. Mus.* **17,** 251–256 (1974).
17. Axsmith, B. J., Taylor, T. N. & Taylor, E. L. Anatomically preserved leaves of the conifer *Notophytum krauselii* (Podocarpaceae) from the Triassic of Antarctica. *Am. J. Bot.* **85,** 704–713 (1998).
18. Axsmith, B. J. & Taylor, T. N. The Triassic conifer seed cone *Glyptolepis*. *Rev. Palaeobot. Palynol.* **96,** 71–79 (1997).
19. Bamford, M. K. Fossil woods of Karoo age deposits in South Africa and Namibia as an aid to biostratigraphical correlation. *J. Afr. Earth Sci.* **31,** 119–132 (1999).
20. Bauer, K. *et al.* Ginkgophytes from the upper Permian of the Bletterbach Gorge (northern Italy). *Riv. Ital. Paleontol. Stratigr.* **120,** 271–279 (2014).
21. Bauer, K., Kustatscher, E. & Krings, M. The ginkgophytes from the German Kupferschiefer (Permian), with considerations on the taxonomic history and use of *Baiera* and *Shenobaiera*. *Bull. Geosci.* **88,** 539–556 (2013).
22. Bercovici, A., Cui, Y., Forel, M.-B., Yu, J. & Vajda, V. Terrestrial paleoenvironment characterization across the Permian–Triassic boundary in South China. *J. Asian Earth Sci.* **98,** 225–246 (2015).

23. Berger, H. A. C. *Die Versteinerungen der Fische und Pflanzen im Sandsteine der Coburger Gegend*. (1832).
24. Berthelin, M., Stolle, E., Kerp, H. & Broutin, J. *Glossopteris anatolica* Archangelsky and Wagner 1983, in a mixed middle Permian flora from the Sultanate of Oman: Comments on the geographical and stratigraphical distribution. *Rev. Palaeobot. Palynol.* **141,** 313–317 (2006).
25. Blanckenhorn, M. Die fossile Flora des Buntsandsteines und des Muschelkalks der Umgebung von Commern. *Paleontographica* **32,** 117–153 (1886).
26. Boersma, M. & Broekmeyer, L. M. Index of figured plant megafossils Triassic 1976–1980. *Rev. Palaeobot. Palynol.* **49,** 235–344 (1986).
27. Bomfleur, B., Krings, M., Kaštovský, J. & Kerp, H. An enigmatic non–marine thalloid organism from the Triassic of East Antarctica. *Rev. Palaeobot. Palynol.* **157,** 317–325 (2009).
28. Borruel-Abadía, V. *et al.* Palaeoenvironmental reconstruction of the early Anisian from sedimentology and plant remains in the SE Iberian Range (E Spain). *Palaeogeogr. Palaeoclimatol. Palaeoecol.* **414,** 352–369 (2014).
29. Brack, P. & Kustatscher, E. *Voltzia recubariensis* from the uppermost Angolo Limestone of the Bagolino succession (Southern Alps of Eastern Lombardy, Italy). *Geo.Alp* **10,** 61–70 (2013).
30. Brea, M., Artabe, A. & Spalletti, L. A. Ecological reconstruction of a mixed Middle Triassic forest from Argentina. *Alcheringa Australas. J. Palaeontol.* **32,** 365–393 (2008).
31. Brongniart, A. T. *Histoire des végétaux fossiles ou recherches botaniques et géologiques sur les végétaux refermés dans les diverses couches du globe. I.* (Dufour et d'Oscagne, 1828).
32. Bronn, H. G. Beiträge zur triasischen Fauna und Flora der bituminösen Schiefer von Raibl, nebst Anhang über die Kurr'sche Sippe *Chiropteris* aus dem Lettekohlen–Sandstein. *N. Jb. Mineral. Geogn. Geol. Petrefactenkunde* **1,** 1–63 (1858).
33. Bronn, H. G. *Lethaea geognostica oder Abbildungen und Beschreibung der für die Gebirgs–Formationen bezeichnendsten Versteinerungen. II. Band Meso–Lethaea: Zweite Periode. Trias Gebirge*. (Schweizerbart'sche Verlagshandlung und Druckerei, 1851).
34. Brown, R. W. New occurrences of the fossil quillworts called *Isoetites*. *J. Wash. Acad. Sci.* **48,** 358–361 (1958).
35. Brunner, H. & Kelber, K. P. Eisenerzkonkretionen im württembergisch–fränkischen Unterkeuper – Bemerkungen zum fossilen Environment. in *Neue Forschungen zur Erdgeschichte von Crailsheim* (ed. Hagdorn, H.) 185–205 (Gesellschaft für Naturkunde in Württemberg, 1988).
36. Burij, J. V. & Zharnikova, N. K. Flora–bearing beds in the Ladinian stage, Middle Triassic, in Southern Primorye. *Byull. Mosk. O-va Ispyt. Prir. Otd. Geol.* **55,** 45–53 (1980).
37. Calligaris, R. Geologia della Val di Braies e segnalazione di nuovo località fossilifere a vegetali nel Ladinico superiore. *Atti Mus. Civ. Storia Nat.* **24,** 1–64 (1986).
38. Calzada, S. Niveles Fosiliferos de la Facies Buntsandstein (Trias) en el Sector norte de los Catalanides. *Cuad. Geol. Iberíca* **11,** 256–271 (1987).
39. Cantrill, D. J. & Webb, J. A. Permineralized pleuromeid lycopsid remains from the Early Triassic Arcadia Formation, Queensland, Australia. *Rev. Palaeobot. Palynol.* **102,** 189–211 (1998).
40. Cariglino, B., Gutiérrez, P. R. & Manassero, M. *Plumsteadia pedicellata* sp. nov.: A new glossopterid fructification from La Golondrina Formation (Guadalupian–Lopingian), Santa Cruz Province, Argentina. *Rev. Palaeobot. Palynol.* **156,** 329–336 (2009).
41. Cassinis, G., Durand, M. & Ronchi, A. Permian–Triassic continental sequences of Northwest Sardinia and South Provence: stratigraphic correlations and palaeogeographical implications. *Boll. Soc. Geol. Ital.* **Volume special 2,** 119–129 (2003).
42. Cassinis, G. & Perotti, C. R. A stratigraphic and tectonic review of the Italian Southern Alpine Permian. *Palaeoworld* **16,** 140–172 (2007).
43. Catullo, T. A. *Memoria Geognostica-Paleozoica sulle Alpi Venete*. (Dai Tipi della R. D. Camera, 1846).

44. Chandra, S., Singh, K. J. & Jha, N. First report of the fertile plant genus *Umkomasia* from late Permian beds in India and its biostratigraphic significance. *Palaeontology* **51,** 817–826 (2008).
45. Charrier, G. Legni fossili del Permiano Medio delle Cuecenes presso Ortisei (Val Gardena). in *La Flora Permiana di Ortisei* (ed. Leonardi, P.) **1,** 19–62 (1964).
46. Chroustchoff, K. von. Ueber einige neue Keuperpflanzen. *Württ. Naturwiss. Jahresh.* **24,** 309–312 (1868).
47. Chu, D. *et al.* Biostratigraphic correlation and mass extinction during the Permian-Triassic transition in terrestrial-marine siliciclastic settings of South China. *Glob. Planet. Change* **146,** 67–88 (2016).
48. Compter, G. Ein Nachtrag zur fossilen Keuperflora Ostthüringens. *Z. Naturwiss.* **86,** 439–447 (1918).
49. Compter, G. Revision der fossilen Keuperflora Ostthüringens. *Z. Naturwiss.* **83,** 81–116 (1911).
50. Compter, G. Cycadeenfrüchte aus der Lettenkohle von Apolda. *Z. Naturwiss.***75,** 169–173 (1902).
51. Compter, G. Die fossile Flora des unteren Keupers von Ostthüringen. *Z. Naturwiss.* **67,** 205–230 (1894).
52. Compter, G. Zur fossilen Flora der Lettenkohle Thüringens. *Z. Naturwiss.* **56,** 1–29 (1883).
53. Cúneo, N. R., Taylor, E. L., Taylor, E. N. & Krings, M. In situ fossil forest from the upper Fremouw Formation (Triassic) of Antarctica: paleoenvironmental setting & paleoclimate analysis. *Palaeogeogr. Palaeoclimatol. Palaeoecol.* **197,** 239–261 (2003).
54. De Zigno, M. E. Sulle piante fossili del Trias di Recoaro raccolte dal Prof. A. Massalongo. *Mem. Ist. Ven. Sc. Lett. Arti* **11,** 1–32 (1862).
55. Decombeix, A.-L., Klavins, S. D., Taylor, E. L. & Taylor, T. N. Seed plant diversity in the Triassic of Antarctica: A new anatomically preserved ovule from the Fremouw Formation. *Rev. Palaeobot. Palynol.* **158,** 272–280 (2010).
56. Decombeix, A.-.-L., Klavins, S. D., Taylor, E. L. & Taylor, T. N. Anatomy and affinities of permineralized gymnospermous trunks with preserved bark from the Middle Triassic of Antarctica. *Rev. Palaeobot. Palynol.* **163,** 26–34 (2010).
57. Diez, J. B. *et al.* Anisian floras from the NE Iberian Peninsula and Balearic Islands: A synthesis. *Rev. Palaeobot. Palynol.* **162,** 522–542 (2010).
58. Diez, J. B. *et al.* Première découverte d'une paléoflore anisienne dans le faciès «Buntsandstein» de la branche ragonaise de la Cordillère Ibérique (Espagne). *CR Acad. Sci. Paris Ser. IIa* **323,** 341–347 (1996).
59. Dobruskina, I. A. *Triassic floras of Eurasia*. **10,** (Österreichische Akademie der Wissenschenschaften, 1994).
60. D'Rozario, A. *et al.* Studies on the Late Permian permineralized tree fern *Psaronius housuoensis* sp. nov. From Yunnan Province, southwest China. **163,** 247–263 (2011).
61. Engel, T. *Geognostischer Wegweiser durch Württemberg. Anleitung zum Erkennen der Schichten und zum Sammeln der Petrefacten*. (Schweitzerbart'sche Verlagshandlung (E. Koch), 1896).
62. Escapa, I. H. *et al.* Triassic floras of Antarctica: Plant diversity and distribution in high paleolatitude communities. *Palaios* **26,** 522–544 (2011).
63. Feng, Z., Wang, J. & Rößler, R. A unique gymnosperm from the latest Permian of China, and ist ecophysiological implications. *Rev. Palaeobot. Palynol.* **165,** 27–40 (2011).
64. Fliche, P. *Flore fossile du Trias en Lorraine et Franche–Compté avec des Considerations finales par M.R. Zeiller.* (Berger-Levrault, 1910).
65. Florin, R. Über *Ortiseia leonardi* n.gen. n.sp., eine Konifere aus den Grödner Schichten in Alto Adige (Südtirol). in *La Flora Permiana di Ortisei* (ed. Leonardi, P.) 3–18 (1964).
66. Franz, M. *et al.* Eustatic and climatic control on the Upper Muschelkalk Sea (late Anisian/Ladinian) in the Central European Basin. *Glob. Planet. Change* **135,** 1–27 (2015).

67. Frentzen, K. Die Pflanzen des Paläozoikums und Mesozoikums. in *Oberrheinischer Fossilkatalog* (ed. Salomon-Calvi, W.) 1–30 (Bornträger Verlag, 1931).
68. Frentzen, K. Die wichtigsten Fundstellen fossiler Pflanzen in Baden und die Entstehung ihrer pflanzenführenden Schichten, 5. Muschelkalk, 6. Keuper, 7. Rhät, 8. Jura, 9. Tertiär. *Badische Geol. Abh.* **1931,** 41–65 (1930).
69. Frentzen, K. Beiträge zur Kenntnis der fossilen Flora des südwestlichen Deutschland. II. Lettenkohlen– und Schilfsandsteinflora. *Jber. Mitt. Oberrh. Geol. Ver.* **11,** 1–14 (1922).
70. Frentzen, K. Die Keurperflora Badens. *Verh. Naturwiss. Ver. Karlsr.* **28,** 1–76 (1922).
71. Freyberg, B. von. Die Keupersammlung Kehl. *Geol. Bl. NO-Bayern* **15,** 151–166 (1965).
72. Fritz, A. & Krainer, K. Eine Rotliegendflora aus Seesedimenten des Bozner Vulkanitkomplexes bei Sinnich (Südtirol). *Geo.Alp* **3,** 33–45 (2006).
73. Fritz, A. & Krainer, K. Eine Rotliegend–Flora aus dem Grödner Sandstein bei Mölten, Südtirol. *Schlern* **73,** 637–650 (1999).
74. Gall, J. C. & Grauvogel-Stamm, L. The early Middle Triassic 'Grès à Voltzia' Formation of eastern France: a model of environmental refugium. *C. R. Palevol* **4,** 637–652 (2005).
75. Gastaldo, R. A. *et al.* Taphonomic trends of macrofloral assemblages across the Permian–Triassic boundary, Karoo Basin, South Africa. *PALAIOS* **20,** 479–497 (2005).
76. Gastaldo, R. A. *et al.* Is the vertebrate-defined Permian-Triassic boundary in the Karoo Basin, South Africa, the terrestrial expression of the end-Permian marine event? *Geology* **43,** 939–942 (2015).
77. Goman'kov, A. V. Floral changes across the Permian-Triassic boundary. *Stratigr. Geol. Correl.* **13,** 74–83 (2005).
78. Grauvogel-Stamm, L. Nouveaux types d'organes reproducteurs mâle de conifères du Grès à Voltzia (Trias inférieur) des Vosges (Grauvogel-Stamm, 1969). Désignation des Holotypes et des Paratypes. *Bull. Serv. Carte Géol. Alsace Lorraine* **22,** 355–357 (1969).
79. Grauvogel-Stamm, L. *La flore du Grès à Voltzia (Buntsandstein supérieur) des Vosges du Nord (France): morphologie, anatomie, interprétations phylogénique et paléogéographique*. (Institut de Géologie, 1978).
80. Grauvogel-Stamm, L. Découverte d'empreintes de conifères dans le Trias de la bordure Nord–Est du massif Central Français. *Sci. Geol. Bull.* **30,** 75–78 (1977).
81. Grauvogel-Stamm, L. & Grauvogel, L. *Aethophyllum* Brongniart 1828, Conifère (non Equisétale) du Grès à Voltzia (Buntsandstain Supérieur) des Vosges (France). *Géobios* **8,** 143–146 (1975).
82. Grauvogel-Stamm, L. & Grauvogel, L. *Masculostrobus acuminatus* nom. nov., un novel organe reproducteur mâle de Gymnosperme du Grès à Voltzia (Trias inférieur) des Vosges (France). *Geobios* **6,** 101–114 (1973).
83. Grauvogel-Stamm, L. & Kustatscher, E. Floren der Germanischen Trias: die Floren des Buntsandsteins und des Muschelkalks. in *Trias – eine andere Welt*
84. Grauvogel-Stamm, L. & Lugardon, B. The Triassic lycopsids *Pleuromeia* and *Annalepis*: Relationships, evolution, & origin. *Am. Fern J.* **91,** 115–149 (2001).
85. Gümbel, C. W. Geognostische Mitteilungen aus den Alpen. V. Die Pflanzenreste führenden Sandsteinschichten von Recoaro. *Sitzungsber. Bayer. Akad. Wiss. Math.-Phys. Cl.* **9,** 33–90 (1879).
86. Haig, D. W. *et al.* Early Triassic (early Olenekian) life in the interior of East Gondwana: mixed marine–terrestrial biota from the Kockatea Shale, Western Australia. *Palaeogeogr. Palaeoclimatol. Palaeoecol.* **417,** 511–533 (2015).
87. Hermsen, E. J., Taylor, E. L. & Taylor, T. N. Morphology and ecology of the Antarcticycas plant. *Rev. Palaeobot. Palynol.* **135,** 108–123 (2009).
88. Holmes, W. B. K. Equisetalean Plant Remains from the Early to Middle Triassic of New South Wales, Australia. *Rec. Aust. Mus.* **53,** 9–20 (1975).
89. Holmes, W. B. K. & Ash, S. R. An Early Triassic Megafossil Flora from the Lorne Basin, N.S.W. *Proc. Linn. Soc. New South Wales Aust.* **103,** 47–70 (1979).

90. Karasev, E. On small pinnate leaves of peltasperm pteridosperms from the Early Triassic of the Kuznetsk Basin (Mal'tsevo Formation, Babii Kamen locality). *Bot. Pacifica* **4,** 131–136 (2015).
91. Kelber, K.-P. Phytostratigraphische Aspekte der Makrofloren des süddeutschen Keupers. *Doc. Naturae* **117,** 89–115 (1998).
92. Kelber, K.-P. Die versunkene Pflanzenwelt aus den Deltasümpfen Mainfrankens vor 230 Millionen Jahren. *Beringeria* **1,** 1–67 (1990).
93. Kelber, K.-P. 5. Die Makroflora des Lettenkeupers. in *Der Lettenkeuper – Ein Fenster in die Zeit vor den Dinosauriern* 51–100 (Staatliches Museum für Naturkunde Stuttgart, 2015).
94. Kelber, K.-P. & Hansch, W. Keuperpflanzen. Die Enträtselung einer über 200 Millionen Jahre alten Flora. *Museo* **11,** 1–157 (1995).
95. Kelber, K.-P. & Nitsch, E. Paläoflora und Ablagerungsräume im unterfränkischen Keuper. *Jber. Mitt. Oberrh. Geol. Ver.* **87,** 217–253 (2005).
96. Kirchner, H. *Die wichtigsten Versteinerungen Frankens*. (Schweitzerbart'sche Verlagsbuchhandlung, 1928).
97. Klebelsberg, R. von. *Geologie von Tirol*. (Gebrüder Borntraeger, 1935).
98. Kustatscher, E. *et al.* Sea-level changes in the Lopingian (late Permian) of the northwestern Tethys and their effects on the terrestrial palaeoenvironments, biota and fossil preservation. *Glob. Planet. Change* **148,** 166–180 (2017).
99. Kustatscher, E. *et al.* Sphenophytes, pteridosperms and possible cycads from the Wuchiapingian (Lopingian, Permian) of Bletterbach (Dolomites, Northern Italy). *Rev. Palaeobot. Palynol.* **208,** 65–82 (2014).
100. Kustatscher, E., Dellantonio, E. & van Konijnenburg-van Cittert, J. H. A. The ferns of the late Ladinian, Middle Triassic flora from Monte Agnello, Dolomites, Italy. *Acta Palaeontol. Pol.* **59,** 741–755 (2014).
101. Kustatscher, E., Franz, M., Heunisch, C., Reich, M. & Wappler, T. Floodplain habitats of braided river systems: depositional environment, flora and fauna of the Solling Formation (Buntsandstein, Lower Triassic) from Bremke and Fürstenberg (Germany). *Palaeobiodiversity Palaeoenvironments* **94,** 237–270 (2014).
102. Kustatscher, E., Bauer, K. & Reich, M. A new Middle Triassic (Pelsonian) plant locality in the Non Valley (Trentino, northern Italy). *Geo.Alp* **9,** 60–73 (2012).
103. Kustatscher, E., Heunisch, C. & van Konijnenburg-van Cittert, J. H. A. Taphonomical implications of the Ladinian megaflora and palynoflora of Thale (Germany). *Palaios* **27,** 753–764 (2012).
104. Kustatscher, E., Kelber, K.-P. & van Konijnenburg-van Cittert, J. H. A. *Danaeopsis* Heer ex Schimper 1869 and its European Triassic species. *Rev. Palaeobot. Palynol.* **183,** 32–49 (2012).
105. Kustatscher, E. *et al.* A new flora from the Upper Permian of Bletterbach (Dolomites, N-Italy). *Rev. Palaeobot. Palynol.* **182,** 1–13 (2012).
106. Kustatscher, E., Giordano, D. & Riva, A. La flora anisica della Valle di San Lucano. in *L'armonia fra uomo e natura nelle Valli Dolomitiche Atti delle giornate di studio, Agordo* (eds. Aldighieri, B. & Testa, B.) 131–146 (Aracne Ed., 2011).
107. Kustatscher, E., Pott, C. & van Konijnenburg-van Cittert, J. H. A. A contribution to the knowledge of the Triassic fern genus *Symopteris*. *Rev. Palaeobot. Palynol.* **165,** 41–60 (2011).
108. Kustatscher, E., Wachtler, M. & van Konijnenburg-van Cittert, J. H. A. Lycophytes from the Middle Triassic (Anisian) locality Kühwiesenkopf (Monta Prà Della Vacca) in the Dolomites (Northern Italy). *Palaeontology* **53,** 595–626 (2010).
109. Kustatscher, E., van Konijnenburg-van Cittert, J. H. A. & Roghi, G. Macrofloras and palynomorphs as possible proxies for palaeoclimatic and palaeoecological studies: A case study from the Pelsonian (Middle Triassic) of Kühwiesenkopf/Monte Prà della Vacca (Olang Dolomites, N-Italy). *Palaeogeogr. Palaeoclimatol. Palaeoecol.* **290,** 71–80 (2010).

110. Kustatscher, E., Hemsley, A. R. & van Konijnenburg-van Cittert, J. H. A. *Lugardonia paradoxa*, a new fertile strobilus with unknown affinities from the Anisian flora of Kühwiesenkopf, the Dolomites, Italy. *Rev. Palaeobot. Palynol.* **156,** 90–97 (2009).
111. Kustatscher, E., Wachtler, M. & van Konijnenburg-van Cittert, J. H. A. Horsetails and seedferns from the Middle Triassic (Anisian) locality Kühwiesenkopf (Monte Prà della Vacca) in the Dolomites (Northern Italy). *Palaeontology* **50,** 1277–1298 (2007).
112. Kustatscher, E., Wachtler, M. & van Konijnenburg-van Cittert, J. H. A. A number of additional and revised taxa from the Ladinian Flora of the Dolomites, Northern Italy. *Geo.Alp* **1,** 57–69 (2004).
113. Kustatscher, E. & van Konijnenburg-van Cittert, J. H. A. Seed ferns from the European Triassic—an overview. in *The Triassic System: New Developments in Stratigraphy and Paleontology* (eds. Tanner, L. H., Spielmann, J. A. & Lucas, S. G.) **61,** 331–344 (2013).
114. Kustatscher, E. & van Konijnenburg-van Cittert, J. H. A. Seed ferns and cycadophytes from the Triassic Flora of Thale (Germany). *N. Jb. Geol. Paläont. Abh.* **258,** 195–217 (2010).
115. Kustatscher, E. & van Konijnenburg-van Cittert. The Ladinian Flora (Middle Triassic) of the Dolomites: Palaeoenvironmental reconstructions and palaeoclimatic considerations. *Geo.Alp.* **2,** 31–51 (2005).
116. Linnell, T. Zur Morphologie und Systematik triassischer Cycadophyten. II. Über Scytophyllum Bornem, eine wenig bekannte Cycadophytengattung aus dem Keuper. *Sven. Bot. Tidskr.* **27,** 310–331 (1933).
117. Linnell, T. Zur Morphologie und Systematik triassischer Cycadophyten I. Die Gattung Sphenozamites Brogniart. *Sven. Bot. Tidskr.* **26,** 241–263 (1932).
118. Mägdefrau, K. Neue Funde fossiler Coniferen im Mittleren Keuper von Haßfurt (Main). *Geol. Bl. NO-Bayern* **3,** 49–57 (1953).
119. McLoughlin, S. New records of leaf galls and arthropod oviposition scars in Permian–Triassic Gondwanan gymnosperms. *Aust. J. Bot.* **59,** 156–169 (2011).
120. McLoughlin, S., Lindström, S. & Drinnan, A. N. Gondwanan floristic and sedimentological trends during the Permian–Triassic transition: new evidence from the Amery Group, northern Prince Charles Mountains, East Antarctica. *Antarct. Sci.* **9,** 281–298 (1997).
121. McLoughlin, S. & Drinnan, A. N. Revised stratigraphy ofthe Permian Bainmedart Coal Measures, northern Prince Charles Mountains, East Antarctica. *Geol. Mag.* **134,** 335–353 (1997).
122. McLoughlin, S. & Drinnan, A. N. Fluvial sedimentology and revised stratigraphy oft he Triassic Flagstone Bench Formation, northern Prince Charles Mountains, East Antarctica. *Geol. Mag.* **136,** 781–806 (1997).
123. Naugolnykh, S. V. *Fossil plants from the Upper Permian of the Cis–Urals (Perm district; collection of G.T. Mauer) at the Vernadsky State Geological Museum*. (2005).
124. Pacyna, G. Plant remains from the Polish Triassic. Present knowledge and future prospects. *Acta Palaeobot.* **54,** 3–33 (2014).
125. Retallack, G. J. The life and times of a Triassic lycopod. *Alcheringa* **1,** 3–29 (1975).
126. Roselt, G. Zur Flora des Unteren und Mittleren Keupers (Dipl. Arbeit 1952). *Wiss. Z. Friedrich–Schiller Univ. Jena Math. Naturwiss. Reihe* **1,** 65–66 (1952).
127. Sadovnikov, G. N. Evolution of the biome of the Middle Siberian Trappean Plateau. *Paleontol. J.* **50,** 518–532 (2016).
128. Sadovnikov, G. N. Paleoecological characterization of the Middle Siberian Trappean plateau during the middle period of its formation (terminal Permian). *Paleontol. J.* **49,** 438–447 (2015).
129. Sadovnikov, G. N. On the global stratotype section and point of the Triassic base. *Stratigr. Geol. Correl.* **16,** 31–46 (2008).
130. Sandberger, F. Die Triasformation im mittleren Maingebiete. *Gem. Wochenschr.* **1–6,** 1–19 (1882).

131. Schaarschmidt, F. & Maubeuge, P. L. Eine männliche Gymnospermen–Fruktifikation aus dem Voltziensandstein (Untere Trias) der Vogesen. *Senckenberg. Lethaea* **50,** 377–397 (1969).
132. Schauroth, K. von. Übersicht der geognostischen Verhältnisse der Gegend von Recoaro im Vicentinischen. *Sitzungsber. Math. Cl.* **17,** 481 (1955).
133. Schenk, A. Bemerkungen über einige Pflanzen der Lettenkohle und des Schilfsandsteines. *Würzbg. Naturwiss. Ztg.* **6,** 49–63 (1867).
134. Schenk, A. Bemerkungen über einige Pflanzen der Keuperformation. *Würzbg. Naturwiss. Ztg.* **3,** 179–180 (1862).
135. Schimper, W. P. & Mougeot, A. *Monographie des Plants fossiles du Grès Bigarré de la chaine des Vosges*. (Chez Guillaume Engelmann, 1844).
136. Schuster, J. *Pagiophyllum weissmanni* im unteren Hauptmuschelkalk von Würzburg. *Geognostische Jahresh.* **1910,** 149–153 (1911).
137. Sennikov, A. G. & Golubev, V. K. Vyazniki biotic assemblage of the terminal Permian. *Paleontol. J.* **40,** S475–S481 (2006).
138. Seyfullah, L. J., Kustatscher, E. & Taylor, W. A. The first discovery of in situ *Verrucosisporites applanatus* spores from the Middle Triassic flora from Bromsgrove (Worcestershire, UK). *Rev. Palaeobot. Palynol.* **197,** 15–25 (2013).
139. Silvestro, D., Cascales-Miñana, B., Bacon, C. D. & Antonelli, A. Revisiting the origin and diversification of vascular plants through a comprehensive Bayesian analysis of the fossil record. *New Phytol.* **207,** 425–436 (2015).
140. Srivastava, A. K. & Agnihotri, D. Dilemma of late Palaeozoic mixed floras in Gondwana. *Palaeogeogr. Palaeoclimatol. Palaeoecol.* **298,** 54–69 (2010).
141. Stockar, R. & Kustatscher, E. The Ladinian flora from the Cassina beds (meride limestone, Monte San Giorgio, Switzerland) – Preliminary results. *Riv. Ital. Paleontol. Stratigr.* **116,** 173–188 (2010).
142. Sun, G., Meng, F., Qian, L. & Ouyang, S. Triassic Floras. in *Fossil floras of China through the geological ages* (eds. Li, X. et al.) 305–410 (Guangdong Science and Technology Press, 1995).
143. Todesco, R., Wachtler, M., Kustatscher, E. & Avanzini, M. Preliminary report on a new vertebrate track and flora site from Piz da Peres (Anisian–Illyrian): Olanger Dolomites, Northern Italy. *Geo.Alp* **5,** 121–137 (2008).
144. Uhl, D. Some considerations on the taphonomy of conifer remains from the Late Permian of Europe. *N. Jb. Geol. Paläont. Mh.* **8,** 483–502 (2006).
145. van Konijnenburg-van Cittert, J. H. A., Kustatscher, E. & Wachtler, M. Middle Triassic (Anisian) Ferns from Kühwiesenkopf (Monte Prá Della Vacca), Dolomites, Northern Italy. *Palaeontology* **49,** 943–968 (2006).
146. Wan, M., Yang, W., Liu, L. & Wang, J. *Ductoagathoxylon jimsarensis* gen. nov. et sp. nov., a gymnospermous stem from the Wuchiapingian (upper Permian) Wutonggou Formation in the Junggar Basin, northern Bogda Mountains, northwestern China. *Rev. Palaeobot. Palynol.* **241,** 13–25 (2017).
147. Wang, Z.-Q. & Wang, L. Late Early Triassic fossil plants in the Shiqianfeng Group in North China. *Shanxi Geol.* **4,** 23–40 (1989).
148. Wang, Z.-Q. & Wang, L. A new species of the lycopsid *Pleuromeia* from the Early Triassic of Shanxi, China, and its ecology. *Palaeontology* **25,** 215–225 (1982).
149. Wang, Z.-Q. & Wang, L. Late Early Triassic fossil plants from upper part of the Shiqianfeng Group in North China. *Shanxi Geol.* **5,** 97–154 (1990).
150. Webb, J. A. A new plant genus, possibly a Marattialean fern, from the Middle Triassic of eastern Australia. *Mem. Australas. Palaeontol.* **1,** 363–371 (1982).
151. Weigelt, J. Neue Pflanzenfunde aus dem Mansfelder Kupferschiefer. *Z. Naturwiss.* **89,** 104–125 (1931).
152. Weigelt, J. Neue Pflanzenfunde aus dem Mansfelder Kupferschiefer. *Leopoldina* **4,** 643–668 (1930).

153. Wills, L. J. On the fossiliferous Lower Keuper rocks of Worcestershire with descriptions of some of the plant and animals discovered therein. *Proc. Geol. Assoc.* **21,** 249–332 (1910).
154. Xiong, C. & Wang, Q. Permian–Triassic land-plant diversity in South China: Was there a mass extinction at the Permian/Triassic boundary? *Paleobiology* **37,** 157–167 (2011).
155. Yu, J., Broutin, J., Huang, Q., Song, Q. & Chen, J. Paleofloral assemblage across the Permian-Triassic boundary, western Guizhou and eastern Yunnan in South China. *J. Earth Sci.* **21,** 179–182 (2010).
156. Yu, J., Broutin, J., Huang, Q. & Grauvogel-Stamm, L. *Annalepis*, a pioneering lycopsid genus in the recovery of the Triassic land flora in South China. *C. R. Palevol* **9,** 479–486 (2010).
157. Yu, J., Li, H., Zhang, S., Yang, F. & Feng, Q. Timing of the terrestrial Permian–Triassic boundary biotic crisis: Implications from U/Pb dating of authigenic zircons. *Sci. China Ser. Earth Sci.* **51,** 1–13 (2008).
158. Yu, J. *et al.* Terrestrial events across the Permian–Triassic boundary along the Yunnan–Guizhou border, SW China. *Glob. Planet. Change* **55,** 193–208 (2007).
159. Zakharov, Y. D. *et al.* Permian to earliest Cretaceous climatic oscillations in the eastern Asian continental margin (Sikhote–Alin area), as indicated by fossils and isotope data. *GFF* **131,** 25–47 (2009).
160. Zamuner, A. B., Zavattieri, A. M., Artabe, A. E. & Morel, E. M. Paleobotánica. in *El Sistema Triásico en la Argentina* (eds. Artabe, A. E., Morel, E. M. & Zamuner, A. B.) **143–184,** 143–184 (Fundacion Museo de la Plata "Francisco Pascasio Moreno", 2001).
161. Zeiller, M. R. Sur la présence, dans le grès bigarré des Vosges, de l'*Acrostichides rhombifolius* Fontaine. in 693–699 (1888).
162. Zhou, Z. & Li, B.-X. A preliminary study of the Early Triassic plants from the Qionghai district, Hainan Island. *Acta Palaeontol. Sin.* **18,** 461–462 (1979).
163. Afonin, S. A. Latest Permian palynological assemblage from Vyazniki, European Russia: stratigraphic and palaeoecological significance in relation to the Permo-Triassic boundary. in *The Nonmarine Permian* (eds. Lucas, S. G. & Zeigler, K. E.) **30,** 5–8 (New Mexico Museum of Natural History and Science, 2005).
164. Afonin, S. A. A palynological assemblage from the transitional Permian-Triassic deposits of European Russia. *Paleontol. J.* **34,** S29–S34 (2000).
165. Antonescu, E. *Ètude de la microflore de l'Anisien de la vallée du Cristian (Braşov)*. **13,** (Institut Géologique, 1970).
166. Balme, B. E. Palynology of Permian and Triassic strata in the Salt Range and Surghar Range, West Pakistan. in *Stratigraphic boundary problems: Permian and Triassic of West Pakistan* (eds. Kummel, B. & Teichert, C.) **4,** 305–453 (University Press of Kansas, 1970).
167. Batten, D. J. & Kovach, W. L. *Catalog of Mesozoic and Tertiary megaspores*. (American Association of Stratigraphic Palynologists Foundation, 1990).
168. Bharadwaj, D. C. The miospore genera in the coals of Raniganj Stage (Upper Permian), India. *Palaeobotanist* **9,** 68–106 (1962).
169. Bharadwaj, D. C., Tiwari, R. S. & Anand-Prakash. Permo-Triassic palynostratigraphy and lithological characteristics in Damodar Basin India. *Biol. Mem.* **4,** 49–82 (1979).
170. Bittniok, B. & Mohr, B. A. Palynologie des Unteren Keupers von Bedheim, Südthüringen. *Veröff. Naturhist. Mus. Schleus.* **17,** 45–60 (2002).
171. Blendinger, E. Palynostratigraphy of the late Ladinian and Carnian in the Southeastern Dolomites. *Rev. Palaeobot. Palynol.* **53,** 329–348 (1988).
172. Broglio Loriga, C. *et al.* The Lower Triassic sequences of the Dolomites (Italy) and Transdanubian mid-mountains (Hungary) and their correlation. *Mem. Sci. Geol.* **42,** 41–103 (1990).
173. Broutin, J., Ferrer, J., Gisbert, J. & Nmila, A. Première découverte d'une microflore thuringienne dans le faciès saxonien de l'île de Minorque (Baléares, Espagne). *C. R. Acad. Sci. Sér. 2 Méc. Phys. Chim. Sci. Univers Sci. Terre* **315,** 117–122 (1992).

174. Brugman, W. A. *A Palynological Characterization of the Upper Scythian and Anisian of the Transdanubian Central Range (Hungary) and the Vicentinian Alps (Italy).* (Rijksuniversiteit te Utrecht, 1986).
175. Brugman, W. A. & Visscher, H. Permian and Triassic palynostratigraphy of northeast Libya. in *Subsurface palynostratigraphy of northeast Libya* (eds. El-Arnauti, A., Owens, B. & Thusu, B.) 157–170 (B. Garyounis University Publications, 1988).
176. Cirilli, S., Pirini Radrizzani, C., Ponton, M. & Radrizzani, S. Stratigraphical and palaeoenvironmental analysis of the Permian-Triassic transition in the Badia Valley (Souther Alps, Italy). *Palaeogeogr. Palaeoclimatol. Palaeoecol.* **138,** 85–113 (1998).
177. Clarke, R. F. A. Keuper miospores from Worcestershire, England. *Palaeontology* **8,** 294–321 (1965).
178. Courtinat, B. & Rio, M. Analyse statistique des palynofacies des depots ladiniens du SE du Massif central francais. *Rev. Micropaléont.* **47,** 28–35 (2004).
179. Dal Corso, J. Palinostratigrafia e stratigrafia integrata dell'Anisico superiore (Triassico) in sezioni scelte delle Alpi meridionali. (Università degli Studi di Padova, 2006).
180. Diéguez, C. & Barrón, E. Late Permian flora and vegetation changes near the Permian–Triassic boundary in the Landete section of the Alcotas Formation (SE Iberian Ranges, Spain). *Palaeogeogr. Palaeoclimatol. Palaeoecol.* **229,** 54–68 (2005).
181. Diez, J. B., Broutin, J. & Ferrer, J. Difficulties encountered in defining the Permian–Triassic boundary in Buntsandstein facies of the western Peritethyan domain based on palynological data. *Palaeogeogr. Palaeoclimatol. Palaeoecol.* **229,** 40–53 (2005).
182. Doubinger, J. & Adloff, M. C. Etudes palynologiques dans le Trias de la bordure sud-est du Massif Central français (Bassin de Largentière, Ardèche). *Sci. Géol. Bull.* **30,** 59–74 (1977).
183. Doubinger, J. & Bühmann, D. Röt bei Borken und bei Schlüchtern (Hessen, Deutschland) Palynologie und Tonmineralogie. *Z. Dtsch. Geol. Ges.* **132,** 421–449 (1981).
184. Doubinger, J., López-Gómez, J. & Arche, A. Pollen and spores from the Permian and Triassic sediments of the Southeastern Iberian ranges, Cueva de Hierro (Cuenca) to Chelva-Manzanera (Valencia-Teruel) region, Spain. *Rev. Palaeobot. Palynol.* **66,** 25–45 (1990).
185. Dridi, M. & Maazaoui, A. *Field Trip Guidebook. Triassic Sandstone Reservoirs in Southern Tunisia. Part I.* **15,** (2000).
186. Eshet, Y. Palynological data on the Zafir Formation, Halal 1 borehole, NE Sinai. *GSI Curr. Res.* **6,** 62–64 (1988).
187. Eshet, Y. *Paleozoic-Mesozoic palynology of Israel: 1. Palynological aspects of the Permo-Triassic succession in the subsurface of Israel.* **81,** (Geological Survey of Israel, 1990).
188. Falcon, R. Palynofloritstic trends in the Permo-Triassic of southern Africa. *IV Int. Palynol. Conf. Lucknow 1976-77* **2,** 208–218 (1980).
189. Farabee, M. J., Taylor, E. L. & Taylor, T. N. Correlation of Permian and Triassic palynomorph assemblages from the central Transantarctic Mountains, Antarctica. *Rev. Palaeobot. Palynol.* **65,** 257–265 (1990).
190. Fijałkowska, A. Palynological aspects of the Permo-Triassic succession in the Holy Cross Mountains, Poland. *Doc. Naturae* **87,** 1–76 (1994).
191. Fijałkowska, A. Palynostratigraphy of the Lower and Middle Buntsandstein in north-western part of the Holy Cross Mts. *Geol. Q.* **38,** 59–96 (1994).
192. Fijałkowska, A. Palynostratigraphy of the Zechstein in the North Sudetic Trough. *Geol. Q.* **39,** 207–228 (1995).
193. Fijałkowska-Mader, A. Palynostratigraphy, palaeoecology and palaeoclimatology of the Triassic in South-Eastern Poland. *Zentralbl. Geol. Paläontol.* **1,** 601–627 (1999).
194. Fisher, M. J. A record of palynomorphs from the Waterstones (Triassic) of Liverpool. *Geol. J.* **8,** 17–22 (1972).
195. Foster, C. B. Permian miospore assemblages from GSQ Taroom 11-11A. *Qld. Gov. Min. J.* **78,** 634–638 (1977).

196. Grauvogel-Stamm, L. & Álvarez Ramis, C. Conifères et pollen in situ du Buntsandstein de l'île de Majorque (Baléares, Espagne). *Cuad. Geol. Ibérica* **20,** 229–244 (1996).
197. Gutiérrez, P. R., Zavattieri, A. M. & Ezpeleta, M. Palynology of the la Veteada Formation (Lopingian) at Its Type Locality, Famatina Range, la Rioja Province, Argentina. Spores. *Ameghiniana* **54,** 441–464 (2017).
198. Hankel, O. Early Triassic plant microfossils from Sakamena sediments of the Majunga Basin, Madagascar. *Rev. Palaeobot. Palynol.* **77,** 213–233 (1993).
199. Hart, G. F. The biostratigraphy of Permian palynofloras. *Geosci. Man* **1,** 89–131 (1970).
200. Helby, R., Morgan, R. & Partridge, A. D. A palynological zonation of the Australian Mesozoic. *Mem. Assoc. Australas. Palaeontol.* **4,** 1–94 (1987).
201. Hermann, E., Hochuli, P. A., Bucher, H. & Roohi, G. Uppermost Permian to Middle Triassic palynology of the Salt Range and Surghar Range, Pakistan. *Rev. Palaeobot. Palynol.* **169,** 61–95 (2012).
202. Heunisch, C. Palynologie des Unteren Keupers in Franken, Süddeutschland. *Palaeontogr. Abt. B* **200,** 33–110 (1986).
203. Hochuli, P. A. & Feist-Burkhardt, S. Angiosperm-like pollen and *Afropollis* from the Middle Triassic (Anisian) of the Germanic Basin (Northern Switzerland). *Front. Plant Sci.* **4,** 1–14 (2013).
204. Jansonius, J. Palynology of Permian and Triassic sediments, Peace river area, western Canada. *Palaeontogr. Abt. B* **110,** 35–98 (1962).
205. Ji, L.-X. & Ouyang, S. Lower Triassic spore-pollen assemblages from the Lower subgroup of Bayanharshan Group, Qinghai. *Acta Palaeontol. Sin.* **45,** 473–493 (2006).
206. Joshi, H., Mishra, S. & Joshi, Y. Palynological Studies in sub-surface sediments from Jangareddygudem area, Chintalapudi sub-basin, Godavari Graben, Andhra Pradesh, India. *Int. J. Geol. Earth Environ. Sci.* **5,** 98–107 (2015).
207. Kar, R. K. Palynological delimitation of the Lower Gondwanas in the North Karanpura sedimentary basin, India. *Palaeobotanist* **20,** 300–317 (1973).
208. Karasev, E. & Turnau, E. Earliest Triassic (Induan) megaspores from Moscow Syneclise, Russia: taxonomy and stratigraphy. *Ann. Soc. Geol. Pol.* **85,** 271–284 (2015).
209. Kilani-Mazraoui, F., Razgallah-Gargouri, S. & Mannai-Tayech, B. The Permo-Triassic of Southern Tunisia — biostratigraphy and palaeoenvironment. *Rev. Palaeobot. Palynol.* **66,** 273–291 (1990).
210. Klaus, W. Sporen aus dem südalpinen Perm (Vergleichsstudie für die Gliederung nordalpiner Salzserien). *Jb. Geol. Bundesanst.* **106,** 229–361 (1963).
211. Koloda, N. A. & Kanev, G. P. Correlation of various facial deposits ofthe Tatarian Stage in the North of European Russia. in *The Nonmarine Permian* (eds. Lucas, S. G. & Zeigler, K. E.) **30,** 151–153 (New Mexico Museum of Natural History and Science, 2005).
212. Krassilov, V. A., Afonin, S. A. & Lozovsky, V. R. Floristic evidence of transitional Permian-Triassic deposits of the Volga-Dvina region. *Permophiles* **34,** 12–14 (1999).
213. Kustatscher, E. & Roghi, G. Anisian palynomorphs from the Dont Formation of the Kühwiesenkopf/Monte Prà Della Vacca Section (Northern Italy). *Micropaleontology* **52,** 223–244 (2006).
214. Lele, K. M. Palaeozoic monosaccate miospores. in *Aspects and Appraisal of Indian Palaeobotany* (eds. Surange, K. P., Lakhanpal, R. N. & Bharadwaj, D. C.) 232–252 (Birbal Sahni Institute of Palaeobotany, 1974).
215. Lindström, S. & McLoughlin, S. Synchronous palynofloristic extinction and recovery after the end-Permian event in the Prince Charles Mountains, Antarctica: Implications for palynofloristic turnover across Gondwana. *Rev. Palaeobot. Palynol.* **145,** 89–122 (2007).
216. Marcinkiewicz, T. The megaspore assemblage of *Capillisporites germanicus* from the Middle Triassic of Poland. *Geol. Q.* **36,** 33–74 (2013).
217. Marcinkiewicz, T., Fijałkowska-Mader, A. & Pieńkowski, G. Poziomy megasporowe epikontynentalnych utworów triasu i jury w Polsce–podsumowanie [Megaspore zones of the

epicontinental Triassic and Jurassic deposits in Poland–overview]. *Biul. Państw. Inst. Geol.* **457,** 15–42 (2014).
218. Massari, F., Neri, C., Pittau, P., Fontana, D. & Stefani, C. Sedimentology, palynostratigraphy and sequence stratigraphy of a continental to shallow-marine rift-related succession: Upper Permian of the eastern Southern Alps (Italy). *Mem. Sci. Geol.* **46,** 119–243 (1994).
219. Murthy, S., Kavali, P. S. & Bernardes-de-Oliveira, M. E. C. Latest Permian palynomorphs from Jharia Coalfield, Damodar Basin, India and their potential for biostratigraphic correlation. *Rev. Micropaléont.* **58,** 167–184 (2015).
220. Ouyang, S. & Utting, J. Palynology of upper Permian and lower Triassic rocks, Meishan, Changxing County, Zhejiang Province, China. *Rev. Palaeobot. Palynol.* **66,** 65–103 (1990).
221. Pereira, Z., Fernandes, P., Lopes, G., Marques, J. & Vasconcelos, L. The Permian–Triassic transition in the Moatize–Minjova Basin, Karoo Supergroup, Mozambique: A palynological perspective. *Rev. Palaeobot. Palynol.* **226,** 1–19 (2016).
222. Prevec, R., Gastaldo, R. A., Neveling, J., Reid, S. B. & Looy, C. V. An autochthonous glossopterid flora with latest Permian palynomorphs and its depositional setting in the *Dicynodon* Assemblage Zone of the southern Karoo Basin, South Africa. *Palaeogeogr. Palaeoclimatol. Palaeoecol.* **292,** 391–408 (2010).
223. Ramos, A. & Doubinger, J. Decouverte d'une microflore thuringienne dans le Buntsandstein de la Cordilliere iberique (Espagne). *C. R. Hebd. Seances Sér. Sci. Nat.* **289,** 525–528 (1979).
224. Roghi, G. Analisi palinologica del Trias medio del Sudalpino. (Università degli Studi di Padova, 1995).
225. Scheuring, B. W. *Palynologische und palynostratigraphische Untersuchungen des Keupers im Bölchentunnel (Solothurner Jura)*. (Birkhäuser Verlag, 1970).
226. Scheuring, B. W. *Mikrofloren aus den Meridekalken des Mte. San Giorgio (Kanton Tessin)*. (Birkhäuser Verlag, 1978).
227. Schneebeli-Hermann, E. *et al.* Vegetation history across the Permian–Triassic boundary in Pakistan (Amb section, Salt Range). *Gondwana Res.* **27,** 911–924 (2015).
228. Schneebeli-Hermann, E., Hochuli, P. A. & Bucher, H. Palynofloral associations before and after the Permian–Triassic mass extinction, Kap Stosch, East Greenland. *Glob. Planet. Change* **155,** 178–195 (2017).
229. Schulz, E. Sporae dispersae aus der Trias von Thüringen. *Mitt. Zent. Geol. Inst.* **1,** 257–287 (1965).
230. Singh, H. P. Saccate pollen grains from the Lower Triassic of Hallstatt, Austria. *Palaeobotanist* **13,** 74–81 (1965).
231. Smith, T. E. & Mantle, D. *Late Permian palynozones and associated CA-IDTIMS dated tuffs from the Bowen Basin, Australia*. **2013/46,** (Geoscience Australia, 2013).
232. Smith, E. G. & Warrington, G. The age and relationships of the Triassic rocks assigned to the lower part of the Keuper in North Nottinghamshire, North-West Lincolnshire and South Yorkshire. *Proc. Yorks. Geol. Soc.* **38,** 201–227 (1970).
233. Steiner, M. B., Eshet, Y., Rampino, M. R. & Schwindt, D. M. Fungal abundance spike and the Permian–Triassic boundary in the Karoo Supergroup (South Africa). *Palaeogeogr. Palaeoclimatol. Palaeoecol.* **194,** 405–414 (2003).
234. Stockar, R., Baumgartner, P. O. & Condon, D. Integrated Ladinian bio-chronostratigraphy and geochrononology of Monte San Giorgio (Southern Alps, Switzerland). *Swiss J. Geosci.* **105,** 85–108 (2012).
235. Surkov, V. S., Kazakov, A. M., Devyatkov, V. P. & Smirnov, V. L. Lower-Middle Triassic rift sequence of the West Siberian basin. *Otechestv. Geol.* **3,** 31–37 (1997).
236. Tewari, R., Mehrotra, N. C., Meena, K. L. & Pillai, S. S. K. Permian megaspores from Kuraloi Area, Ib-River coalfield, Mahanadi Basin, Orissa. *J. Geol. Soc. India* **74,** 669–678 (2009).
237. Tiwari, R. S. Palaeozoic cryptogamic spores. in *Aspects and Appraisal of Indian Palaeobotany* (eds. Surange, K. P., Lakhanpal, R. N. & Bharadwaj, D. C.) 84–99 (Birbal Sahni Institute of Palaeobotany, 1974).

238. Tiwari, R. S. & Tripathi, A. A report of Raniganj mioflora from sediments of Dubrajpur Formation in Brahmani Coalfield, Rajmahal Basin. *Geophytology* **14,** 244–245 (1984).
239. Tiwari, R. S. & Tripathi, A. Marker Assemblage-Zones of spores and pollen species through Gondwana Palaeozoic and Mesozoic sequence in India. *Palaeobotanist* **40,** 199–236 (1992).
240. Tripathi, A., Vijaya, Murthy, S., Chakarborty, B. & Das, D. K. Stratigraphic status of coal horizon in Tatapani–Ramkola Coalfield, Chhattisgarh, India. *J. Earth Syst. Sci.* **121,** 537–557 (2012).
241. Tripathi, A., Vijaya & Raychowdhuri, A. K. Triassic palynoflora from the Mahuli-Mahersop Area, Singrauli Coalfield (southern extension), Sarguja District, Chhattisgarh, India. *J. Palaeontol. Soc. India* **50,** 77–99 (2005).
242. Van de Laar, J. G. M. *Aspects of Upper Permian and Lower Triassic Palynology: Palynological Investigations in the Upper Permian and Lower Triassic of the Vicentinian Alps and Western Dolomites (NE-Italy)*. (Laboratory of Palaeobotany and Palynology, State University Utrecht, 1980).
243. Van der Eem, J. G. L. A. Aspects of Middle and Late Triassic palynology. 6. Palynological investigations in the Ladinian and lower Karnian of the Western Dolomites, Italy. *Rev. Palaeobot. Palynol.* **39,** 189–300 (1983).
244. Vigran, J. O., Mangerud, G., Mørk, A., Worsley, D. & Hochuli, P. A. *Palynology and geology of the Triassic succession of Svalbard and the Barents Sea*. (Geological Survey of Norway, 2014).
245. Wang, M.-Z., Zhang, X.-Q. & Zhao, X.-L. Palynological assemblages of Upper and Lower Shihezi formations of Permian from western Shandong region and the basal boundary of Upper Shihezi Formation. *Acta Palaeontol. Sin.* **43,** 349–365 (2004).
246. Yang, J. *et al. Permian and Triassic strata and fossil assemblages in the Dalongkou area of Jimsar, Xinjiang*. **3,** (Geological Publishing House, 1986).
247. Yao, Z. & Shu, O. *On the Paleophyte-Mesophyte boundary.* (Nanjing Institute of Geology and Paleontology, 1980).
248. Yaroshenko, O. P. Triassic miospore assemblages from the western Caucasus. *Proc. IV Int. Palynol. Conf. Lucknow 1976-77* **2,** 254–260 (1980).
249. Zhang, K. *et al.* Early Triassic conodont–palynological biostratigraphy of the Meishan D Section in Changxing, Zhejiang Province, South China. *Palaeogeogr. Palaeoclimatol. Palaeoecol.* **252,** 4–23 (2007).
