# Supplementary material for: No mass extinction for land plants at the Permian–Triassic transition
Source: Nat Commun. 2019 Jan 23;10:384. doi: 10.1038/s41467-018-07945-w (PMC6344494; doi:10.1038/s41467-018-07945-w)
Supplement: Supplementary file 3 — Description of Additional Supplementary Files [file 41467_2018_7945_MOESM3_ESM.pdf]

Description of Additional Supplementary Files

File Name: Supplementary Data 1
Description: Range charts of plant macro- and microfossil taxa across the Wuchiapingian to Ladinian interval.

File Name: Supplementary Data 2
Description: Summarizing the calculated plant diversity indices.

File Name: Supplementary Data 3
Description: Spearman‘s rank correlation coefficient for the correlation between selected diversity indices and corresponding p-values.

File Name: Supplementary Data 4
Description: Raw occurrence data of land plant macrofossils, with taxonomic and stratigraphic corrections.

File Name: Supplementary Data 5
Description: Raw occurrence data of spores and pollen, with taxonomic and stratigraphic corrections.

File Name: Supplementary Data 6
Description: R script for the generation of the results presented in Supplementary Data 1–3 from the data given in Supplementary Data 4 and 5.
